# Spontaneous Dipole Reorientation in Confined Water and Its Effect on Wetting/Dewetting of Hydrophobic Nanopores

**DOI:** 10.1021/acsami.3c17272

**Published:** 2024-02-01

**Authors:** Yuriy G. Bushuev, Yaroslav Grosu, Mirosław Chorążewski

**Affiliations:** † Institute of Chemistry, University of Silesia in Katowice, Szkolna 9 Street, 40-006 Katowice, Poland; ‡ Centre for Cooperative Research on Alternative Energies (CIC EnergiGUNE), Basque Research and Technology Alliance (BRTA), Alava Technology Park, Albert Einstein 48, Vitoria, Gasteiz 01510, Spain

**Keywords:** nanoporous materials, hydrophobic nanotubes, pure silica zeolites, intrusion/extrusion, hydrogen-bonded network

## Abstract

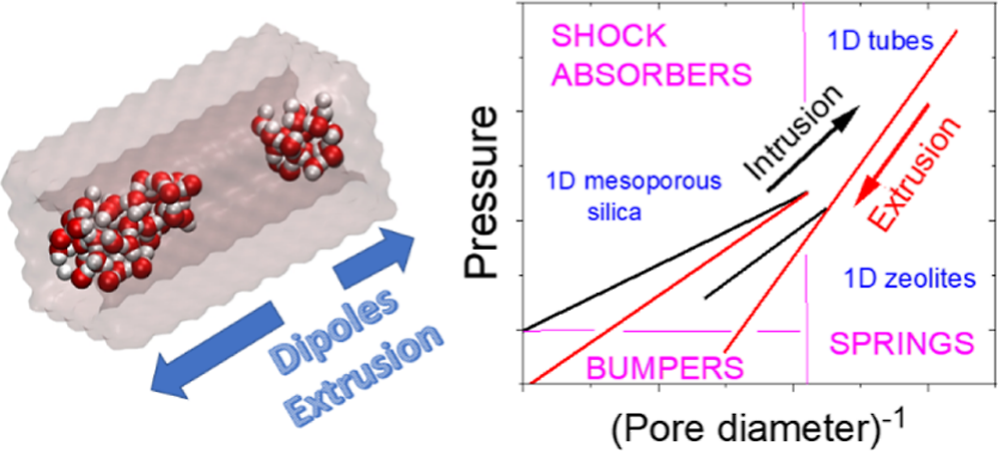

The properties of nanoconfined fluids are important for a broad range of natural and engineering systems. In particular, wetting/dewetting of hydrophobic nanoporous materials is crucial due to their broad applicability for molecular separation and liquid purification; energy storage, conversion, recuperation, and dissipation; for catalysis, chromatography, and so on. In this work, a rapid, orchestrated, and spontaneous dipole reorientation was observed in hydrophobic nanotubes of various pore sizes *d* (7.9–16.5 Å) via simulations. This phenomenon leads to the fragmentation of water clusters in the narrow nanopores (*d* = 7.9, 10 Å) and strongly affects dewetting through cluster repulsion. The cavitation in these pores has an electrostatic origin. The dependence of hydrogen-bonded network properties on the tube aperture is obtained and is used to explain wetting (intrusion)–dewetting (extrusion) hysteresis. Computer simulations and experimental data demonstrate that *d* equals ca. 12.5 Å is a threshold between a nonhysteretic (spring) behavior, where intrusion–extrusion is reversible, and a hysteretic one (shock absorber), where hysteresis is prominent. This work suggests that water clustering and the electrostatic nature of cavitation are important factors that can be effectively exploited for controlling the wetting/dewetting of nanoporous materials.

## Introduction

Nanoconfinement brings unexpected and sometimes counterintuitive properties in liquids, with water being the most fascinating example.^1−3^ Understanding nanoconfined liquids is of paramount importance for a broad range of technologies, including purification, nanolubrication, separation, and energy. In particular, heterogeneous lyophobic systems (HLSs) consisting of porous solids and nonwetting liquids have many applications, including chromatography, molecular separations, energy storage, energy conversion, recuperation, dissipation, and so on.^4−11^ Zeolites, metal–organic and covalent organic frameworks (MOFs, COFs), carbon nanotubes (CNTs), mesoporous silica, and porous liquids are natural and synthetic porous materials that have been intensively studied and applied.^12−18^ Heterogeneous hydrophobic systems (HHSs) involving hydrophobic porous matrices and water have attracted much attention due to their simplicity and abundance. Moreover, wetting/dewetting processes are vital in biology and nanoscience, where water usually interacts with hydrophobic materials and structures.^19−21^

The hydrophobicity, pore opening size, geometry, topology of a pore system, and pore morphology are key parameters that determine the wetting of porous materials. A plethora of studies was devoted to investigating moderately hydrophobic and wetted ambient-pressure CNTs.^16,22^ A single-walled CNT is a 1D cylindrical tube with a circular cross section that makes it favorable for modeling and computer simulations of heterogeneous water–CNT systems. It was shown that water changes its properties under confinement, and many anomalies were found. For example, new phases of ice, not observed for bulk water, are formed at high pressures.^22,23^

For more hydrophobic materials, water can be intruded into pores only at an elevated pressure. Intrusion–extrusion isotherms, the *P*–*V* diagrams, characterize this process. Extrusion can be observed at the same or lower pressure or may not occur at all. These HLSs are called molecular spring, shock-absorber, and bumper, respectively.^7,10^

Pure silica zeolites (PSZs) have larger hydrophobicity compared to that of CNTs, and water cannot permeate into crystals at ambient pressure. Plenty of zeolite topologies, pore sizes, pore shapes, and mutual intersections of porous channels hinder the systematic investigation of their wetting/dewetting. The chemical instability of some PSZs makes the situation even more complicated. Silanol defects of frameworks can be formed after water intrusion. They significantly decrease the hydrophobicity of PSZs, influencing extrusion pressure and, thus, the system behavior.^7,24,25^ Progressive degradation of materials during the cyclization of intrusion–extrusion processes prevents their applications in some technologies.

Mesoporous silica materials are amorphous and hydrophilic due to silanol groups covering internal pore surfaces. They receive considerable attention due to their simple production and applications for medical diagnostics or drug delivery systems.^26,27^ Using the methods of silane chemistry, they can be easily functionalized and made hydrophobic. However, synthesis conditions and the procedure of functionalization affect the pore size and volume and introduce heterogeneities at the nanoscale. The water intrusion–extrusion isotherms were obtained for some grafted mesoporous materials with the 1D system of channels.^28−30^

The dimensionality of the pore system affects water intrusion–extrusion. It was shown that hydrophobicity is regulated not only by the chemical modification of internal pore surfaces but also by closing lateral pores.^31,32^ Computer simulations and correlations of experimental intrusion pressures with the ratio of Connolly surface area to pore volume show that PSZs with 1D channels have larger intrusion pressure than zeolites with other topologies with the same pore-opening diameters.

Despite intensive investigations,^15,33−35^ the peculiarities of intermolecular interactions, molecular mechanisms of processes, and the structure of liquids under nanoconfinement are not well established. Many natural factors affect the wetting/dewetting processes in HHSs. The main goal of this work is to systematically investigate the collective behavior, dynamical state, structure, and interactions of water under different nanoconfinements and their effect on the wetting/dewetting process. The observed kinetics of molecular populations of tubes and processes of dipole reorientations indicate new factors for controlling the wetting/dewetting hysteresis of hydrophobic nanopores.

## Results

**Figure 1:**
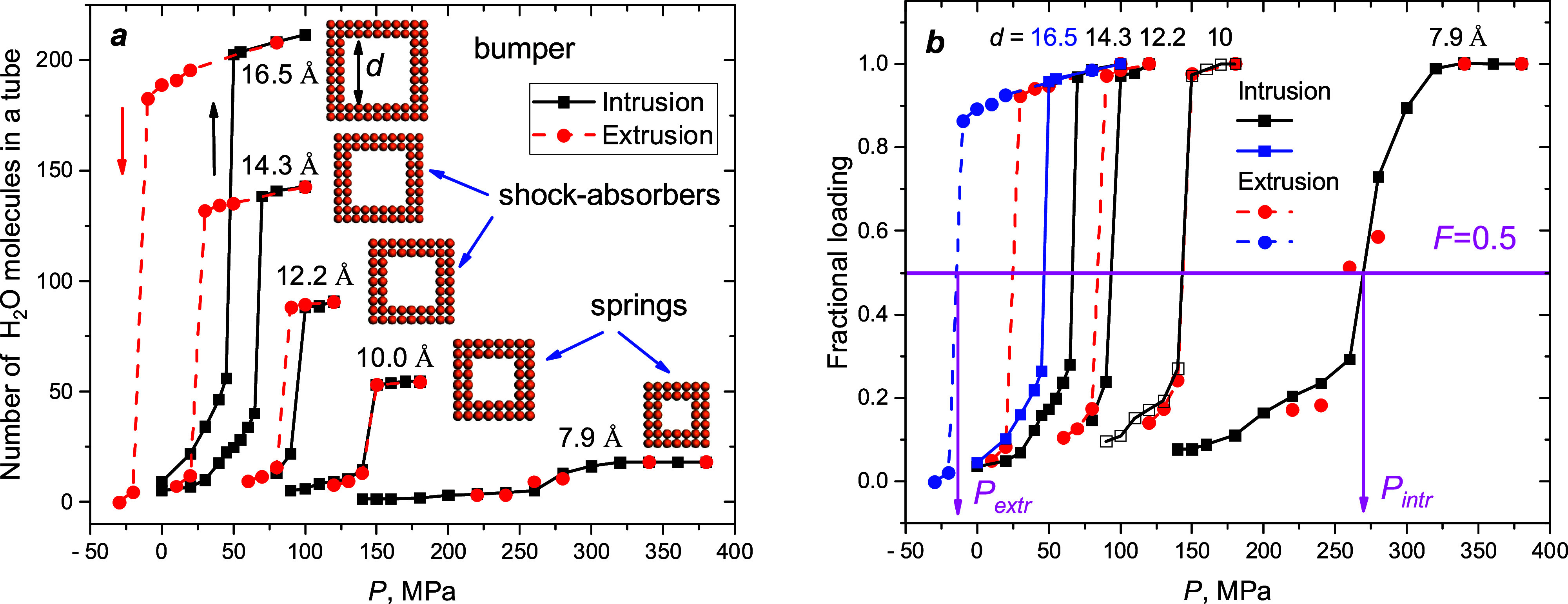
Intrusion–extrusion isotherms demonstrate three types of behavior for systems with different pore apertures: (a) number of water molecules vs pressure; cross sections of the pores are presented in insets; (b) fractional loadings vs pressure. *P*_intr_ and *P*_extr_ correspond to the half-loading of the tubes by definition.

Intrusion–extrusion isotherms are the main results of experimental investigations of wetting and dewetting for HHSs. The isotherms, calculated for five nanotubes and presented in Figure 1, demonstrate pore loading versus external hydrostatic pressure. Two types of data presentations are exploited in the present work. The number of molecules in the tubes, *N*(*P*), is proportional to the adsorbed water volume. Meanwhile, the fractional loadings *F*(*P*) = *N*(*P*)/*N*_max_ highlight curve differences and allow determining intrusion–extrusion pressures (*P*_intr_, *P*_extr_), which correspond to *F* = 0.5 by definition. Depending on the pore aperture, the three behaviors of heterogeneous systems are detected in our study: bumper (*P*_extr_ < 0), shock-absorber (*P*_extr_ < *P*_intr_), and spring (*P*_extr_ = *P*_intr_).

Atomistic simulations allow the intrusion–extrusion process to be observed step by step. Small water clusters, the groups connected by hydrogen-bond molecules, enter the tube from both sides. With the increasing pressure, they propagate at larger distances and coalesce afterward if the pressure is larger than *P*_intr_. However, as will be shown further, the dynamic behavior of water clusters after intrusion and during extrusion depends on the pore width.

Two slopes appear on the curves in Figure 1. The first region corresponds to growing clusters with pressure, which occupy more and more room in tubes. The fast transition from a partially to totally loaded state is due to the relatively short tubes (ca. 39 Å). In an actual experimental situation, the pore length is orders of magnitude longer than that in our cases, and the experimental isotherms are smooth functions of pressure. However, the isotherms calculated for the narrowest pore are shallow-sloped, and they correspond to specific processes and water structures in the tube described below.

**Figure 2:**
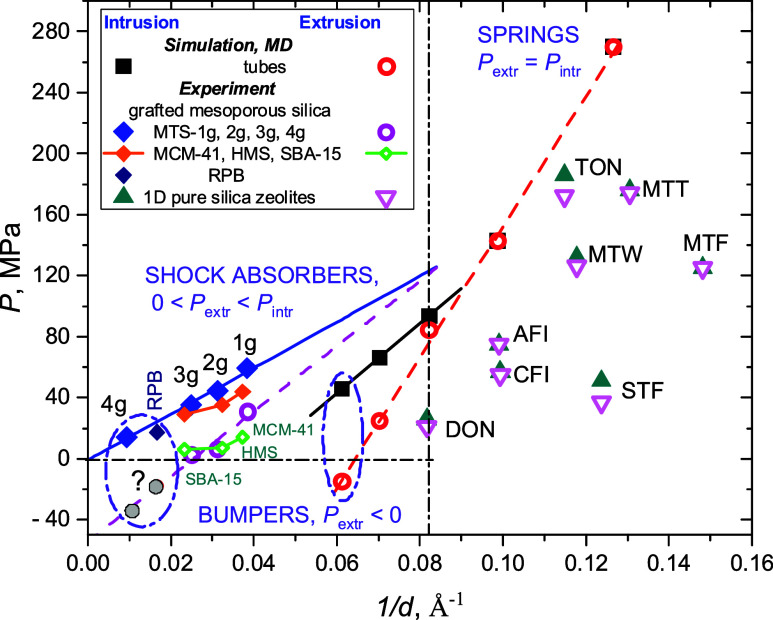
Intrusion–extrusion pressures vs reverse pore apertures for tubes, PSZs, and mesoporous silica. Estimated extrusion pressures are shown by gray circles.

Figure 2 demonstrates the correlations of intrusion–extrusion pressures with the reversed pore diameter, *d*^–1^. This correlation was chosen according to the Laplace–Washburn equation that defines the capillary pressure as
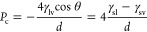
1where γ_lv_, γ_sl_, and γ_sv_ are liquid–vapor, solid–liquid, and solid–vapor surface tension, respectively; θ is the contact angle; and *d* is the pore diameter. In our cases, it is difficult to define surface tension due to the extreme nanoconfinement because several water molecules are on the tips of the propagating clusters. However, the linear correlations of intrusion–extrusion pressures with *d*^–1^ are observed for the investigated systems.

The computational results are compared with the experimental ones obtained for PSZs^36^ and grafted amorphous mesoporous silica materials with the 1D system of channels.^28−30^ This topology was selected because the intrusion pressures significantly depend on the dimension of a pore.^31^Table 1S contains all experimental data presented in Figure 2, whereas Figure S1 demonstrates the zeolite pore aperture measurement method.

The hydrophobicity of materials depends on their quality and grafting density as well as functional groups. Grafted MCM-41, HMS, and RPB are less hydrophobic than MTS materials because the intrusion pressure is smaller than that expected from the correlation line calculated for MTS data. We used the MTS data for the correlation because they were obtained for the same material and method of grafting.^28^ The quality of materials, the grafting protocol, and the material’s morphology affect the pressures.^30^

The external surface of PSZs has a hydrophobicity that is very different from that of the internal surface. This difference is due to the presence of hydrophilic OH groups.^37,38^ Estimating the hydrophobicity of the channels and pores of zeolites using standard experimental methods is not possible.^39^ Intrusion pressure correlates with the hydrophobicity of materials. However, pore shapes and water–wall interactions depend on the material and grafting procedure. The data presented in Figure 2 show that the hydrophobicity of simulated tubes is higher than the hydrophobicity of PSZs but lower than that of grafted mesoporous silica. At the same pore aperture (for example, at 1/*d* = 0.82 Å^–1^), intrusion pressure increases in the order: PZS, tubes, mesoporous silica.

The data for the 1D PSZs are listed on the right side of Figure 2. Previously, it was shown that all PSZs can be divided into three groups depending on their topology and the ratio of the Connolly surface to the free volume of pores.^31^ The dimension, the shape of pores, and chemical interactions of silica with water affect the pressures.^24,25^ Some defects in the crystalline structure usually appear after water loading. Silanol groups, forming hydrophilic nests, decrease hydrophobicity and, as a result, extrusion pressure. Thus, small hysteresis is observed for PSZs with 1D channels, but generally, zeolites demonstrate a spring behavior. For PSZs, the correlation with *d*^–1^ is monotonic and follows a similar trend as for the tubes and grafted silica. However, it is noticeably poorer due to some uncertainties in *d* definition due to a noncircular shape of the pore’s cross sections.^40^

The difference between intrusion and extrusion pressures is hysteresis. For the tubes, after a certain threshold, hysteresis appears and increases with the size of the pore aperture. Extrapolation of intrusion/extrusion fitting lines obtained for the MTS shows their intersection in the region very close to the intersection of the lines obtained for the tubes. Consistently, zeolites are all located in the spring region of *d*. All of the materials have the 1D system of channels, but hydrophobicity is different. It explains the different slopes of the fitting lines. The hydrophobicity of tubes is higher than that of PSZs but smaller than that of grafted mesoporous silica. However, we highlight the geometrical effect here: the wider the pore aperture—the larger the hysteresis. The estimated spring or shock-absorber behavior threshold is ca. 12.5. Å.

The largest tube and two mesoporous silica materials (MTS-4g and RPB) demonstrate the bumper behavior, *P*_extr_ < 0. After intrusion, water stays in pores even at ambient pressure. The estimated extrusion pressures are shown by gray circles in Figure 2. Large negative pressures are unreachable in actual experiments.

**Figure 3:**
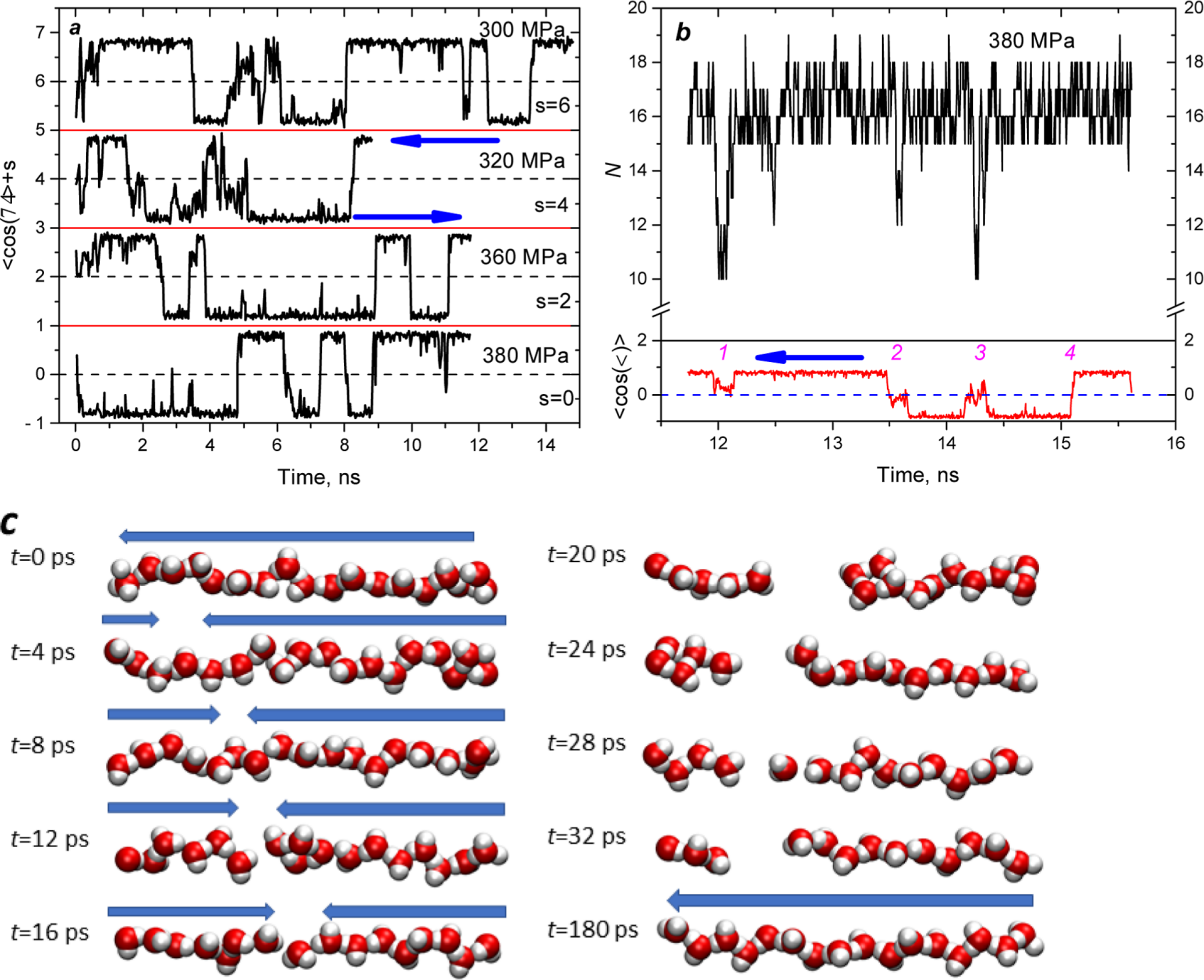
Time evolution of the average cosine between dipole moments and the axial direction of the tube (*d* = 7.9 Å): (a) at different pressures; (b) at *P* = 380 MPa. (c) Water clusters in the tube corresponding to event no. 1. Arrows show the orientation of dipole moments.

Thus, computer models and experiments show three behaviors of HHSs composed of porous materials with a 1D system of channels. The width of pores and the hydrophobicity of materials determine the behavior. In our simulations, two narrow tubes are molecular springs. The structure of confined water was investigated to understand the obtained trend of hysteresis with the tube size.

### Tube with *d* = 7.9 Å

First, we discuss the water structure and its dynamic behavior for the tube with the narrowest pore. Hydrogen-bonded water molecules form a single-file structure—a 1D chain.^41^ Molecular dipole moments in H-bonded chains can be collectively “left-” or “right-side” oriented. For each saved molecular configuration, we calculated the average cosine angle α between the dipole moment of the molecules in the tube and the axial axis. The results are presented in Figure 3a. Most of the time, all molecular dipoles are preferentially oriented toward one side of the tube. However, in nanosecond intervals at any pressure larger than *P*_intr_, several events corresponding to the fast dipole reorientation are observed. The hops between states often occur during 40 ps, evidencing collective molecular reorientation.

To study the process in detail, we calculated at *P* = 380 MPa the average cosine concentration and the number of molecules in the tube for the configurations saved with a time step of 4 ps. Four reorientation events are visible in Figure 3b. The first one occurs near 12 ns, with a duration of about 180 ps. Here, the collective dipole reorientations are accompanied by a partial depletion of the tube. The corresponding molecular configurations of water in the tube are shown in Figure 3c. During 16 ps, the reorientation wave propagated along the tube at a speed of 100 m/s (1 Å/ps). After that, the cluster loses connectivity, and two separated clusters with the “head-to-head” orientation and empty space between them are formed. Finally, the initial single-file structure has been restored. The same process is observed in Figure 3b at 14.2 ns (event no. 3). However, event no. 4 is different. Reorientation of the dipole moments is not accompanied by dewetting of the tube. The snapshots of the corresponding molecular configurations are shown in Figure S2. Again, the speed of the reorientation wave is about 100 m/s. During event no. 2, all dipoles changed the initial orientation, and empty space formation was observed.

At a smaller pressure (*P* = 320 MPa), the partial and total dewetting of the tube was observed approximately at 2 and 4 ns, respectively, as presented in Figure S3. These events are synchronized with the changes in the average cosine, which are close to zero due to the oppositely directed dipoles in the two clusters. With decreased pressure, the empty space increases due to the rise in the distance between clusters. There are no water molecules in the empty spaces. Repulsive electrostatic interactions create an additional internal pressure that increases the probability of dewetting.

Thus, two water clusters, formed after dipole-reorientation-induced splitting, can shrink, grow, or merge, restoring the single-file structure. In computer simulations, the lengths of tubes are orders of magnitude shorter than those in materials used for actual experiments. For long channels, fragmentation of water is expected. Empty spaces are formed between oppositely oriented water clusters. Under a higher pressure, the dynamic equilibrium shifts toward the more filled state of the channel, making isotherms shallow-sloped. Fragmentation is the factor that provokes additional internal resistance to water penetration. Experiments support this speculation. Shallow-sloped isotherms are observed for PSZs with narrow 1D channels, having MTT,^42^ MTW, and TON^43^ topologies. This has great importance for intrusion-based porosimetry techniques, including water intrusion porosimetry.^44^ Typically, shallow-sloped isotherms are attributed to porous materials with a broad pore size distribution. However, our results suggest that the slope of the intrusion isotherm has nothing to do with the pore size distribution; rather, it is related to an additional energetic penalty due to electrostatic interactions of oppositely oriented dipoles of water in the nanopore.^45^

### Tube with *d* = 10 Å

**Figure 4:**
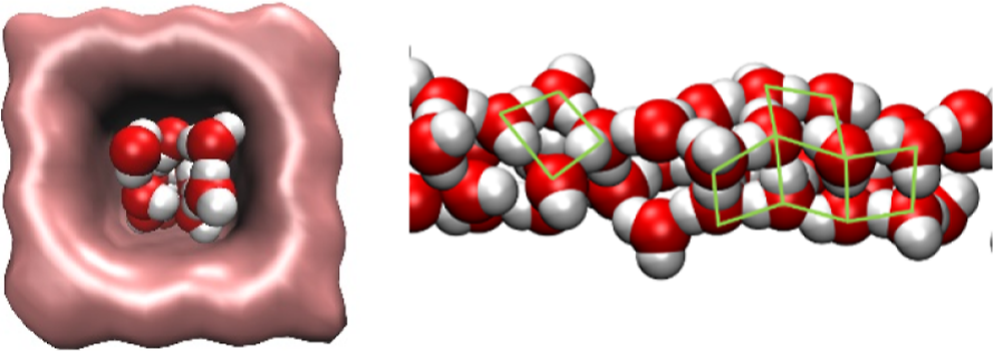
Water clusters are formed in the tube with *d* = 10 Å. Green lines highlight the four-membered rings of H bonds.

The geometry of the pores affects the shape of the water cluster, as presented in Figure 4. Four molecules are visible in the cross section, and the partially disordered square ice structure with four-membered rings (MRs) elements is observed (Figure S4). The formation of ice phases with different structures is a well-known phenomenon of water nanoconfinement.^2,22^ Cylindrical nanotubes, mostly CNTs, were the objects for investigations. Phase diagrams of water were calculated for CNTs with *d* = 11.1 and 12.5 Å.^23^ Depending on the pressure and temperature, five ice phases were identified. Square ice is formed in the narrow tube.

In contrast to CNTs, there are no straight cylindrical channels among the zeolite topologies. We observed a liquid behavior in the tube. The associated four MR fragments can be embryos of the solid phase. Near the solid–liquid coexistence line, nucleation can be a very slow process, taking time far exceeding the time for computer simulations. The phenomenon of ice–liquid oscillations observed at nanoconfinement is more probable.^2^Figure S4 demonstrates spontaneous ice formation in almost the whole tube, but the ice melts with time. The absence of wetting/dewetting (intrusion–extrusion) hysteresis in experiments indicates the liquid state of water in the PSZs. Otherwise, ice would clog channels.

**Figure 5:**
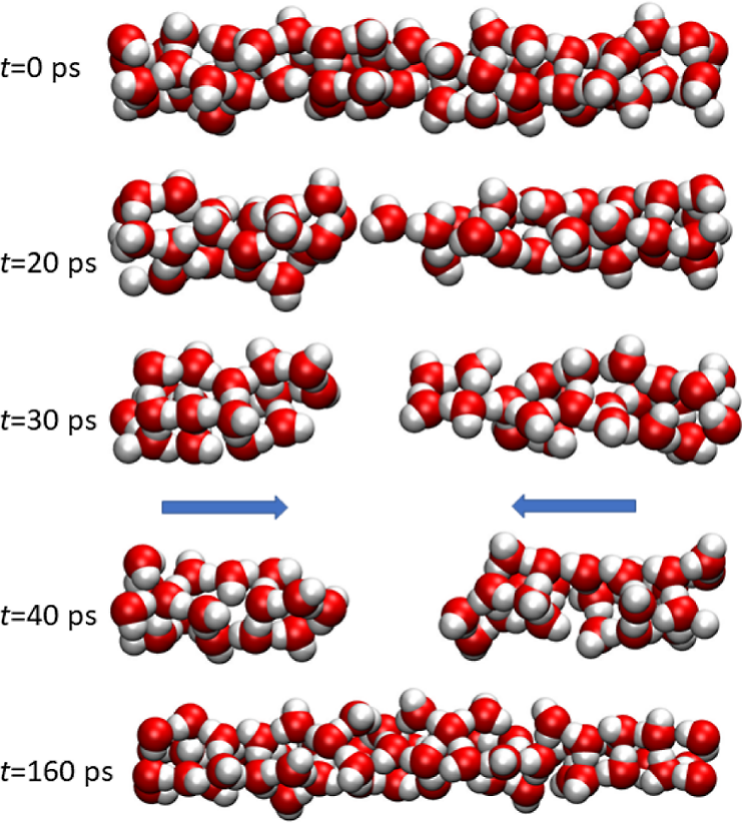
Water clusters in the tube with *d* = 10 Å. Arrows show the preferential orientation of the dipole moments.

The average cosine fluctuates in time with a smaller amplitude than in the case of the tube with *d* = 7.9 Å because several interconnected chains of water molecules with oppositely directed dipoles are formed (see Figure S5). However, a spontaneous splitting of water into two clusters is observed. Two events are presented in Figures 5 and S6. Again, electrostatic interactions between clusters prevent their coalescence over hundreds of picoseconds. However, we do not observe a complete depletion of the tube at *P* > *P*_intr_.

We may imagine the water cluster as four interconnected rubber threads to simplify the picture and better understand the process of cluster splitting. Unlike in a single-file structure, dipole reorientation must occur in four chains simultaneously. The probability of this event is rather small. Figure S7 demonstrates clusters that are close to being split. One can see a break of two or three threads, but the elasticity of the remaining threads is enough to prevent the complete disconnection and partial depletion of the tube in these cases. It is expected that spontaneous dewetting at *P* > *P*_intr_ is a rare event, but water fragmentation occurs in long channels. Again, dipole–dipole repulsion is responsible for splitting.

### Tubes with *d* > 12 Å

**Figure 6:**
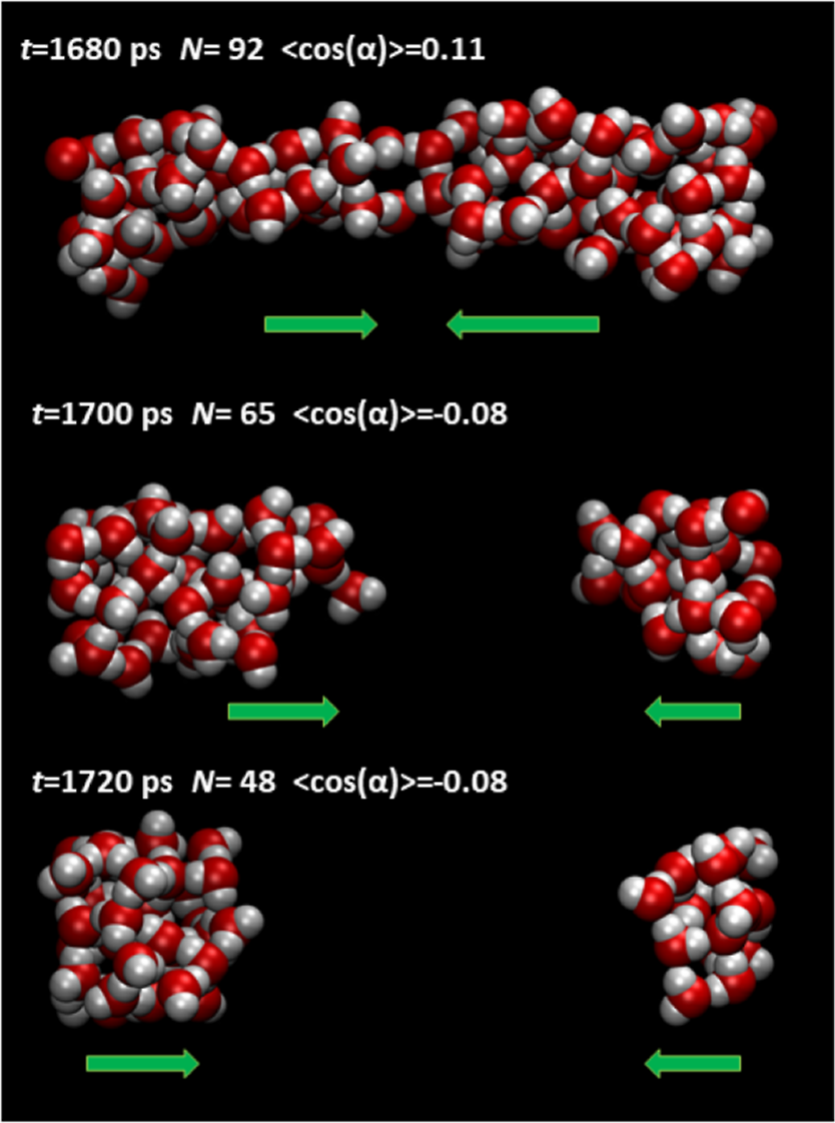
Extrusion of water from the tube with *d* = 14.3 Å at *P* = 10 MPa. Arrows show the preferential orientation of dipole moments, *t* is the time mark, and *N* is the number of water molecules in the tube.

Intrusion–extrusion hysteresis is observed for these tubes, but at *P* > *P*_intr_, there are no breaks of percolated clusters. Several extrusion events are presented in Figure 6 and S8–S10. Average cosines are closer to zero than that in previously discussed cases due to the summation of differently oriented dipoles. Meanwhile, note that the extrusion process has stages: a local decrease in molecular density; formation of chains bridging bulky domains with oppositely directed dipoles; splitting of the cluster; and fast expulsion of fragments. Dipole–dipole interactions force the depletion of tubes.

### Spontaneous Dipole Reorientation as a Triggering Factor for Dewetting (Extrusion)

The collective dipole reorientation described above provides a molecular level of understanding of the dewetting process of hydrophobic nanopores. Spontaneous cavitation is observed in molecular configurations at *P* < *P*_extr_, but molecules still form clusters. There is no vapor phase in tubes at the explored temperature. The formation of bridges between bulky fragments and the breaking of “rubber threads” testify to the spinodal decomposition mechanism when metastable water loses mechanical stability. Fast and orchestrated orientational jumps provoke the formation of mutually repulsive domains connected by bridges. However, the bridges are the fragments of water structures observed in narrower pores that expel water at a higher pressure. The threads suddenly lose elasticity due to dipole reorientation. Thus, fast spontaneous molecular reorientation is a governing factor for dewetting.

We have observed only one mechanism of tube dewetting. Some events are presented in Figures 6 and S8–S10. However, cluster splitting can occur due to large spontaneous density or energy fluctuations, but the probability of such events is small. In the ensemble of channels in actual materials, their main part is dewetted through the orchestrated dipole reorientation of water molecules.

### Properties of H-Bonded Networks

How does microporous confinement affect the water structure? It is a challenging question. We performed a set of calculations to answer it. The ability to form hydrogen bonds is one of the crucial properties dictating the structure of water. Many definitions of H bonds are used to analyze computed molecular configurations.^46^ In this work, we apply the threshold criterion, considering two molecules bonded if the energy of their interactions is less than −3.5 kcal/mol (*E*_HB_ < −14.64 kJ/mol), a typical energy of the middle-strength H-bond. It was shown^47,48^ that the uncertainty in the definition of H bonds does not principally influence the final qualitative results. Scanning the water H-bonded network’s properties using the different *E*_HB_ values gives additional information about weak and strong H-bond distributions.

The topological properties of the H-bonded networks were analyzed: the statistics of molecules with different connectivities—the fractions of molecules forming *i* bonds with neighbors (*i* = 0–4)^47^ and the statistics of closed rings of H bonds in the network.^48−50^ We compared the properties of water in the tubes with the corresponding properties of bulk water and water in imaginary tubes—the bulk water in the cutoff spaces with the geometrical parameters of the actual tubes. Water in imaginary tubes is in ideal hydrophilic surroundings. Molecules form hydrogen bonds with other water molecules through imaginary borders. However, the topological properties of H-bonded networks differ from those of bulk water due to artificial confinement, which depends on the tube size. Thus, a comparison of water properties for water in actual and imaginary tubes and bulk water highlights the confinement and the hydrophobicity effects.

The first characteristic under consideration is the statistics of H bonds. If each bond forms with probability *p* and breaks with the probability (1 – *p*), then in the model of the random network, the fraction of molecules with *i* bonds is defined as^47^
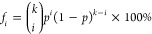
2

Here, we assume that a water molecule can form four bonds at the maximum; thus, *k* = 4. The probability is calculated as *p* = *n*_HB_/4, where *n*_HB_ is the mean number of H bonds per molecule, which depends on the H-bond definition. Some molecules in a molecular ensemble form bifurcated H bonds. We counted molecules with more than four bonds as four-bonded to compare the calculated fractions with the theoretical distribution, eq 2.

The second characteristic is ring statistics. A closed ring consists of molecules connected by hydrogen bonds if the path along the bonds comes back to the starting molecule. We searched all rings for each molecule until six-membered rings (6 MRs), not only short-circuited as it is usually adopted.^51^ Thus, the same molecule can belong to several rings, not only the primitive ones. Our method has been used previously to investigate the topology of H-bonded networks formed in several liquids.^48−50^ The closed ring statistics are characteristic of the supramolecular structure. Crystalline structures contain a specific set of rings. Hexagonal rings (6 MRs) are the basis of ice Ih and Ic. Five and six MRs are the elements of the network in clathrate hydrates. All types of rings are found in bulk water, but we will discuss only 3–6 MRs for simplicity.

**Figure 7:**
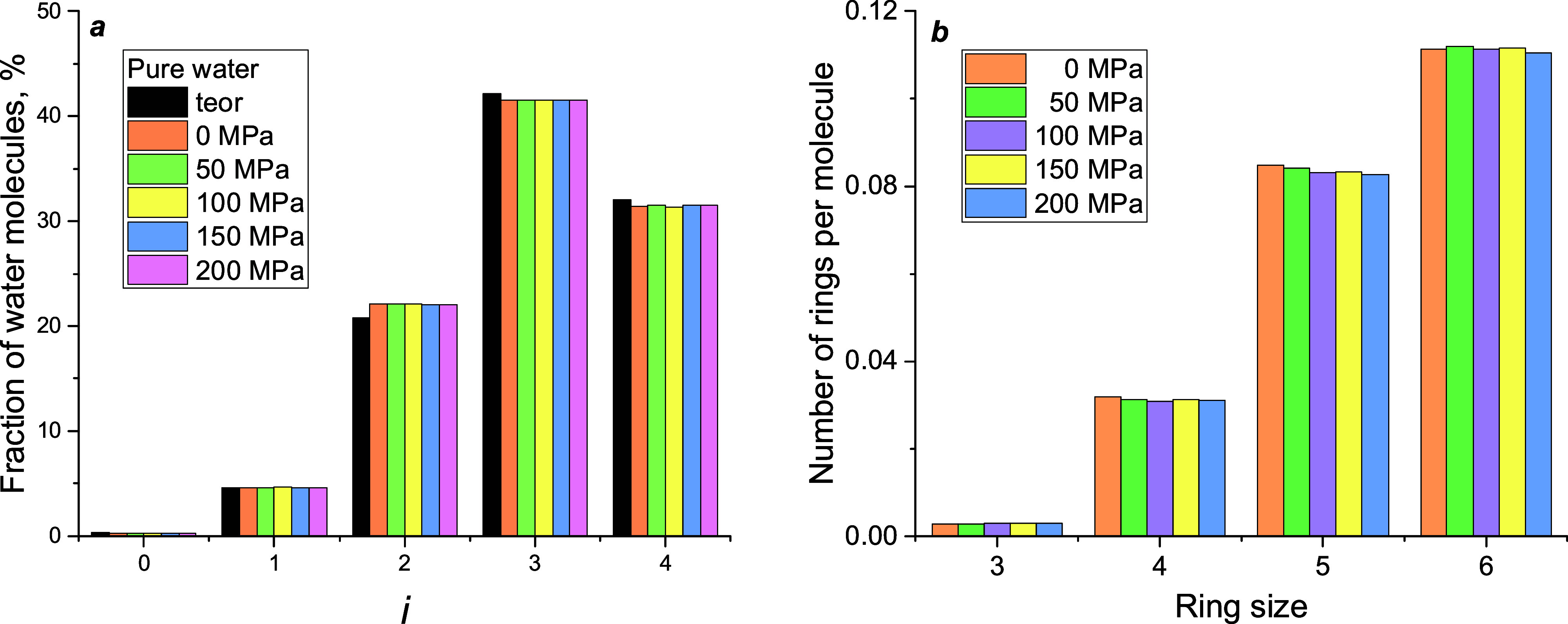
Statistics of H bonds (a) and rings (b) for bulk water at different pressures. Here, *i* is the number of hydrogen bonds in which a molecule participates.

Bulk water is the reference system for comparison to the properties of confined water. Calculations show that the mean number of H bonds per molecule is 3.01, and it does not depend on the external pressure. The distribution of molecules versus the number of H bonds they participated and the ring statistics are presented in Figure 7. We conclude that pressure minimally affects these properties of the network at the accepted definitions of H bond and pressure range. The thermodynamic properties of water, such as volume or enthalpy, depend on pressure. We consider only relatively strong H bonds forming a skeleton of the network with high elasticity. Pressure deforms these H bonds but does not break them. Only small deviations from the theoretical predictions (eq 2) are observed in Figure 7a. Thus, H bonds are formed randomly and distorted only under pressure. It reminiscences the well-known and widely considered random network model of water.^52^

### Water is Confined in Tubes

**Figure 8:**
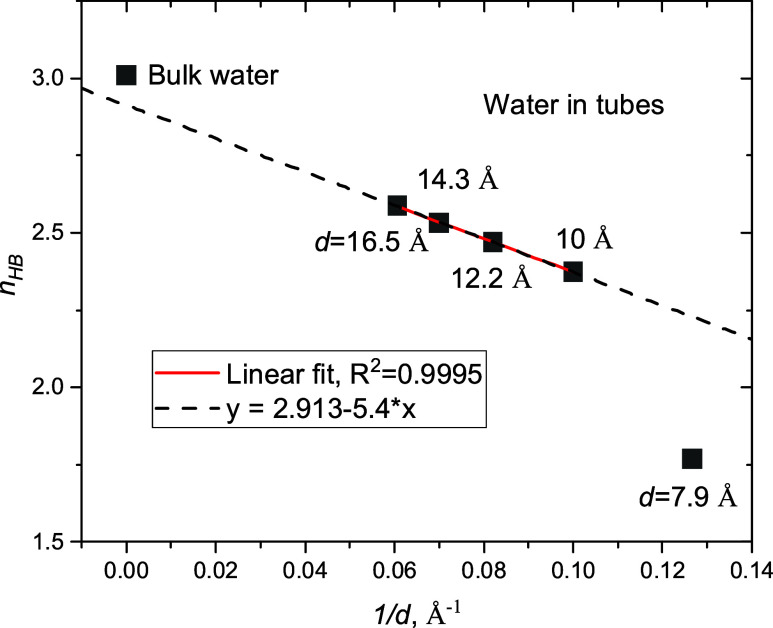
Mean number of H bonds in which a water molecule participated. Data for four tubes were linearly fitted (red line) and extrapolated (dashed black line).

The mean number of H bonds per water molecule decreases with a decrease in the pore width, as presented in Figure 8, evidencing network disruption. A linear correlation with *d*^–1^ is observed for four tubes, but if the bulk water is not too far from the extrapolated value, water in the narrowest tube is out of the correlation. In the perfect single-file structure, each molecule participates in two H bonds. The calculated *n*_HB_ < 2 corresponds to the formation of separated water clusters in this tube.

**Figure 9:**
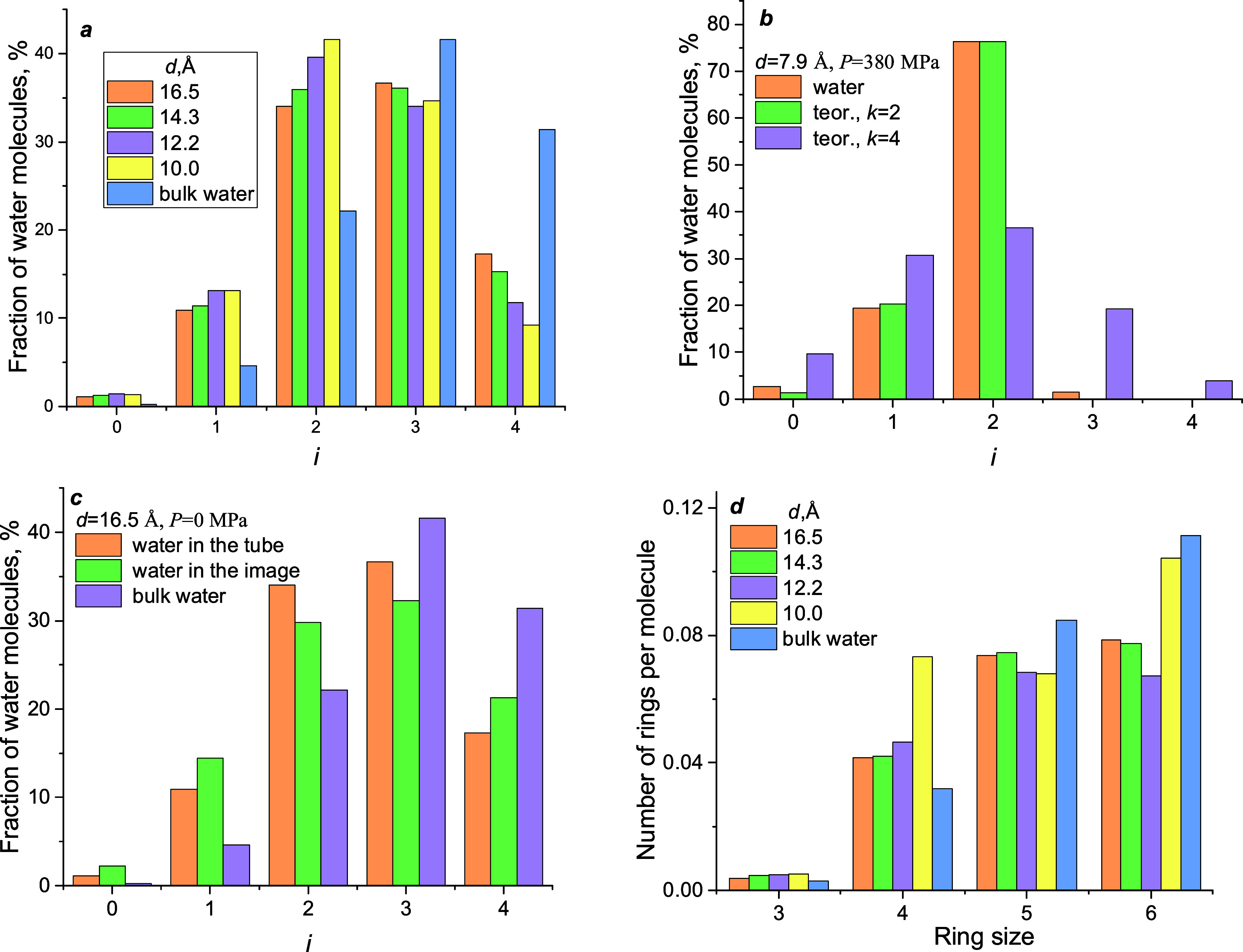
Statistics of H bonds for water: (a) in tubes with *d* ≥ 10 Å; (b) in the tube with *d* = 7.9 Å, comparing with theoretical predictions at *k* = 2 and 4, eq 2; (c) in the tube with *d* = 16.5 Å, comparing with bulk water and water in the imaginary tube. (d) Statistics of closed rings for bulk water and water in tubes.

Statistics of *f*_*i*_ for water molecules in tubes with *d* > 8 Å are presented in Figure 9a. The fraction of molecules with four bonds (*i* = 4) progressively decreases, but *f*_2_ progressively increases with the narrowing of pores. Fractions of other types of water molecules do not vary significantly. Two factors affect these changes: a decrease in *n*_HB_ and the alteration of the water structure due to confinement. Molecules near tube surfaces cannot form some H bonds due to sterical obstacles. It leads to an increase in their potential energy. The network adopts a new configuration to minimize this effect. Interconnected chains and square ice structures are the typical fragments in the networks, especially at *d* = 10 Å.

Are H bonds formed randomly in the tubes? To answer this question, we compared the statistics of bonds with the theoretical prediction in eq 2. Histograms presented in Figure S11 demonstrate an increase in the fractions of molecules with two Hbonds (*i* = 2) and a decrease in *f*_4_. The changes progressively increase with the degree of confinement. The distribution drastically changes for the narrowest pore (*d* = 7.9 Å), as shown in Figure 9b, and far from the distribution predicted by eq 2 with *k* = 4, but if we assume that *k* = 2, *f*_*i*_ will correspond to the theoretical values. It means water molecules predominately form two H bonds instead of four, but they again are formed randomly with the probability *p* = *n*_HB_/2. Thus, we see the smooth distortion of the water structure in a specific range of pore openings until the critical value *d* < 10 Å. After that, the structure changes abruptly. However, H bonds are formed randomly in all cases.

Finally, we calculated the statistics for water molecules occluded in imaginary tubes in bulk water (Figure 9c). Only molecules whose coordinates correspond to the geometrical parameters of the internal space of tubes were selected in bulk water molecular configurations. We consider some H bonds broken because they link molecules across imaginary boundaries. These are the molecules forming H bonds with “ideal hydrophilic walls”. Thus, the effect of hydrophobic walls is highlighted. One can see three different distributions in Figure 9c. Due to confinement, the fractions of molecules with three and four H bonds decrease while *f*_0_ – *f*_2_ increase. For really occluded water, fractions of molecules with two and three bonds increase with respect to “imaginary confined” water. Considering a reduction of *f*_0_ and *f*_1_, we may conclude that the network distortion in the hydrophobic tube tends to be minimal. If molecules cannot form hydrogen bonds with walls, they minimize potential energy, forming additional bonds with molecules in the tube.

Statistics of rings are listed in Figure 9d. Again, as in the case of H-bond statistics, the confined water structure is significantly distorted compared with bulk water. The numbers of 5 and 6 MRs decrease, while the numbers of topological elements corresponding to distorted water structures (3 and 4 MRs) increase. Square cross sections of tubes stimulate the formation of 4 MRs, but the number of 4 MRs progressively decreases with the tube diameter. There is no significant difference in the distributions for the large tubes (*d* = 14.3 and 16.5 Å), but they are still far from bulk water. Confinement suppresses the formation of 6 MRs in the networks, but for the tube with *d* = 10 Å, the increase in 4 MRs is accompanied by an increase in 6 MRs. This evidences the formation of a partially disordered square ice structure because 6 MRs can be represented as a couple of 4 MRs with a shared edge (see Figure 4).

Does the tube shape affect the formation of square ice? Computer simulations demonstrate that this ice is formed in cylindrical CNTs,^23^ silicon carbide nanotubes,^53^ and irregularly shaped cylindrical channels of AFI-type PSZ.^54^ These investigations show that ice formation has a general character and that the type of ice depends on the width of the pore and, to a small extent, on the chemistry or pore shape.

The study of nucleation and crystallization demands long productive runs reaching a few microseconds.^23^ This investigation was not our primary task. Each crystal structure has a specific set of topological elements of the network––the “fingerprints”. According to our calculations, water in tubes is in a disordered state. It does not contradict ice–liquid oscillations observed in molecular configurations (Figure S4). The spring behavior of water–PSZ systems with 1D channels provided compelling evidence of the liquid state of confined water. In all tubes, liquid water is observed, but its structure is significantly altered due to a decrease in the average number of H bonds with narrowing and specific confinement and hydrophobic effects.

## Discussion

Our results demonstrate a strong effect of spontaneous collective dipole reorientation of nanoconfined water on the wetting/dewetting of hydrophobic nanopores. In particular, we demonstrate that the contact of two formed water clusters with oppositely oriented dipoles triggers dewetting (extrusion) due to electrostatic repulsion.

For bulk water, it was shown^55^ that groups of molecules spontaneously, abruptly, and collectively change their orientations during a subpicosecond time, in other words, in the THz time scale. The fast and orchestrated angular jumps are predominantly located in the patches of the H-bonded network with a lower density and many defects. Confinement provokes such a behavior. In the narrowest tube, molecules in the chain form two H bonds with neighbors. The spontaneously broken H bond due to the angular jump of one molecule induces orchestrated reorientations of dipole moments in large clusters and, as a result, increases the probability of dewetting of a hydrophobic nanopore. In particular, our simulations show that dipole reorientations in the tube of 39 Å length and diameter of 7.9 Å occur during 40–200 ps, corresponding to the GHz range. The partial reorientation stimulates chain splitting. At hydrophobic tubes under elevated pressure, electrostatic repulsion of formed clusters with oppositely directed dipole moments creates an additional internal pressure, hindering their coalescence that facilitates dewetting (extrusion).

Our simulations were performed for tubes whose lengths are shorter than the channels in zeolite crystals but more prolonged than the length of biological nanopores.^56^ The size of continuous clusters with the unimodal orientation of dipole moments depends on the temperature, pressure, hydrophobicity, width, and length of channels. In micrometer-sized channels, we expect water to form clusters when groups of molecules are separated by voids, which is usually called the vapor phase. However, the volume fraction of the voids is small and decreases with pressure (see Figure 1). A dipole–dipole repulsion strongly depends on distance. Figures 3c and 5 demonstrate typical voids, whose lengths are approximately 4–7 Å.

Because of the state above, one may expect that the wetting (intrusion)/dewetting (extrusion) pressure depends on an external electric field, which inhibits spontaneous dipole reorientations and promotes wetting. An electrowetting effect was observed^56,57^ in the computer simulations of short pores (ca. 10–30 Å) with lower hydrophobicity than the hydrophobicity of PSZs and grafted mesoporous silica. The voltage-gated ion channel mechanism is crucial in understanding the nature of biological membrane permeability.^56,58^

Charged particles on the outer and inner surfaces can influence porous materials’ wetting/dewetting properties. In this article, we discuss only PSZs that do not contain any extra-charged particles like aluminum atoms in the framework and extra-framework counterions. Even a small amount of Al decreases both pressures due to the strong interactions of water molecules with ions and the formation of water clusters.^55,59^ The same effect was observed for the silanol defects.^60^ The synthesis and methods of PSZs and mesoporous materials characterization are described in the literature.^28−30,61^ Deionized and degassed water was used for intrusion–extrusion experiments. Thus, experimentally studied systems could contain only trace amounts of ions.

Intrusion–extrusion experiments with PSZs show that both pressures shift toward high values when aqueous salt solutions are used instead of water.^10^ Ions, adsorbed on the outer surface of micrometer-sized crystals, cannot affect pressures due to charge screening at long distances from the surfaces where most pores are situated. Ions can affect the wetting/dewetting of porous materials if they penetrate pores. The increase in osmotic pressure and partial desolvation of hydrated ions can explain the increase in intrusion/extrusion pressures. The ion dehydration was detected experimentally.^62,63^ It has been shown that intruded species are ions coordinated by a smaller number of water molecules than those in the bulk solution. On the other hand, PSZs are exploited to produce reverse osmosis membranes used for water desalination.^64^

Monte Carlo^65^ and MD^66^ simulations of narrow water-filled CNTs demonstrate that a static electric field of ca. 10^8^ V/m decreases the diffusion coefficient of water in CNTs while increasing the average number of H bonds. For hydrophobic tubes with an aperture diameter of 20–30 Å, MD simulations showed that the same electric field does decrease the extrusion pressure.^67^

The main problem is the achievement of such a strong field in experiments. An isolated monovalent ion in a vacuum creates an electric field of ca. 10^8^ V/m at a distance of less than 20 Å, and it decreases fast with the distance. The static dielectric permittivity of a liquid (ε) proportionally reduces the field. Electric screening effects manifest in nanotubes.^41,68^ Meanwhile, the simulations of a single-file structure reaction on the isolated ion imposed in a vacuum near the CNT wall demonstrate the soliton-like propagation of dipole reorientation with the speed of 720 m/s.^69^ It is 1 order of magnitude faster than in our case of spontaneous reorientation and defect diffusion through the hydrophobic tube under elevated pressure. Cavitation hinders soliton propagation over long distances. An electric field is expected to provoke the soliton mechanism and support proton transport along 1D chains^41^ formed in pores under pressure.

The experimental evidence of the intrusion pressure decrease due to external electric field was obtained for ZSM-5 (MFI) zeolite.^70^ This zeolite has a 3D network of narrow channels. Therefore, at any moment in the zeolite–water dispersion, only a small part of water molecules in micropores are oriented along the applied electric field. However, intrusion pressure decreased by 15% with the increase in strength of the electric field to 2400 V/m, which is 5 orders of magnitude weaker than the field used in computer simulations. This effect may be due to the inhibition of the spontaneous dipole reorientation of nanoconfined water.

Thus, experiments and computer simulations show that ions or static electric fields affect the wetting/dewetting of hydrophobic pores. Suppression of orchestrated dipole reorientation explains the electrowetting effect. Additional computer simulations are needed to investigate how a static electric field affects the frequency of reorientations, water cluster sizes, and the structure of water.

We hypothesize that a more effective control of wetting/dewetting can be obtained by applying microwave electromagnetic radiation. The main idea is that water in the tube is in an altered state. It differs from bulk water, and we may expect different frequencies of dipole reorientations. The oscillation of the electric field vector in resonance with collective dipole reorientation may provoke fast energy absorption and the transformation of energy into heat.

This hypothesis is supported by the previous computations of the effect of microwaves on water in narrow CNTs.^71^ The maximum of the imaginary part of complex-frequency-dependent dielectric permittivity, responsible for dielectric losses and microwave absorption, is shifted toward the lower frequency range with respect to bulk water, where the maximum is at ca. 20 GHz. According to calculations, the maximum microwave absorption for (8,8) CNT with the single-file water structure is observed near 2.45 GHz, the frequency used in ordinary microwave ovens, where water is relatively transparent, and the reflection of waves from walls provides their deep penetration into a heating object and effective absorption.

Suppose a gap separates the microwave absorption bands for confined and bulk water. In that case, one can control intrusion–extrusion pressures and heat release in a porous material by applying microwave electromagnetic radiation. Additional pressure must be applied to suppress chain fragmentation and water boiling inside pores. Hence, intrusion–extrusion pressures can be controlled by the power or direction of radiation in the case of the anisotropic orientation of pores. The pores should be oriented along the electromagnetic wave propagation direction to maximize the effect of the electric field on the intrusion–extrusion pressure. Therefore, membranes with ordered pores are preferred for this strategy. On the contrary, powderous materials, where channels are randomly oriented, should have a lower effect of an electric field that is expected to result in shallow-sloped isotherms. The same can be stated about the 2D and 3D pore systems.

### Heated Water in Micropores Expels from Hydrophobic Pores at a High Speed

It was proposed to exploit this effect for the engineering of nanorockets.^72^ The possible construction of the nanorocket using water heating inside double-walled CNTs was demonstrated. One of the applications of nanorockets is drug delivery vehicles.^73^ If our hypothesis is correct, the design of the nanorocket may be simple, and pulsating microwave radiation used as the energy source will help the cyclization of the process and permanent motion of nanoparticles. The new propulsion mechanism can be used widely.^73,74^

Thus, the observed effects for nanoconfined water under pressure stimulate new theoretical and experimental investigations that promise a broad range of prospective applications covering energy storage, liquid purification, the production of nanoparticles with unique properties, and many more.

## Conclusions

It was demonstrated that the collective dipole reorientation mechanism in liquid water is manifested under nanoconfinement and plays a crucial role in the system’s wetting/dewetting behavior. At elevated pressures, we found the instability of the single-file structure observed in the narrowest hydrophobic pore under investigation (*d* = 7.9 Å) due to the fast and orchestrated reorientation of molecular dipoles, followed by splitting and repulsion of formed water clusters. The wave of sequaciously jumping dipoles propagates the tube with a speed of ca. 100 m/s. As a result, the total dipole reorientation in a molecular chain occurs, or the chain splits into fragments, which increases the probability of dewetting (extrusion) due to the repulsive electrostatic interactions of oppositely oriented dipoles in separated molecular chains that create the internal pressure responsible for cavitation.

The proposed mechanism plays a significant role in wider tubes (*d* = 12.2–16.5 Å) during water expulsion. With a decrease in pressure (*P* < *P*_intr_), water goes into the stretched and metastable state, followed by the loss of mechanical stability. A splitting water cluster is stabilized by chain bridging, while it is destabilized by electrostatic interactions and the spontaneous reorientation of dipoles.

By combining the simulation and experimental data for PSZs, grafted mesoporous silica, and tubes, we demonstrate that the wetting (intrusion)–dewetting (extrusion) hysteresis appears for pore apertures larger than ca. 12.5 Å and progressively increases with the width of the pore.

Statistics of H bonds and closed rings were analyzed to characterize the water structure under confinement. It was demonstrated that the bulk water structure is altered in pores. The mean number of H bonds per molecule decreases linearly with *d*^–1^ for all pores except the narrowest, manifesting progressive disruption of the H-bonded network. In the meantime, the H-bond statistics generally correspond to the theoretical predictions assuming random formation and breaking of bonds. The water structure is abruptly changed in the narrowest pore, where a single-file structure is observed. The analysis of statistics of closed rings of H bonds, which characterize the topological properties of networks, has shown that the square shape of the pore’s cross sections stimulates the formation of 4 MRs. Embryos of the square ice structure—the associated 4 MRs—are observed in the pore with *d* = 10 Å.

Tubes with pore openings of 7.9 and 10 Å demonstrate the molecular spring behavior. The water structure is the most distorted due to confinement in these tubes. With the increase in the tube diameter, the structure gets closer to the structure of bulk water, and water stays in tubes at a pressure lower than intrusion pressure due to its ability to be in the metastable state.

The obtained results are useful for designing heterogeneous systems for energy storage and absorption and explaining a broad range of phenomena where water interacts with porous hydrophobic materials, such as liquid purification, separation, catalysis, and so on. We expect that dipole reorientation jumps under confinement are sensitive to the electric field and microwave radiation, which can be used to regulate the wetting/dewetting of pores and to be among the dominant factors controlling the hydrophobicity of porous materials. Additional investigations must be performed to elucidate this hypothesis.

## Methods

We investigated simplified systems to highlight the role of the size of the pore openings. Porous materials were made from the face-centered cubic (fcc) crystal structure of gold. Five tubes of different widths and the same length were prepared. The tubes were excavated from the crystals so that the wall thickness was the same for all tubes; an example is presented in Figure S12.

In contrast with the cylindrical single-walled CNTs, the tailored tubes have square cross sections and thick walls that make them closer to real systems, such as zeolites. To generalize the results of computations, we modeled systems whose properties are close to those of both PSZs and some mesoporous silica materials for which experimental measurements were done.

We define the internal size of a tube *d* as the distance between the centers of atoms. It equals the edge of the square cross section. Otherwise, not well-defined distances may be assigned due to the “softness” of atoms. It is a source of additional uncertainties. Tubes were tailored from atoms with Lennard-Jones parameters of water oxygens; thus, the accessible size of a pore is about 2.8 Å less than *d*. Oxygens cover the zeolite pore surfaces. It is difficult to assign an average parameter for pore opening because of the irregular shape of pores in 1D channel systems (see Figure S1).^40^ The definition of pore aperture for mesoporous silica materials is not as critical as in the case of microporous ones, and we took them from the original publications.^28^

In our models, the pore openings *d* were 7.9, 10, 12.2, 14.3, and 16.5 Å, whereas the length of the tubes was 39 Å. The tubes were immersed in orthorhombic boxes with 20,000 water molecules. Periodic boundary conditions were applied to eliminate surface effects. The Melchionna modification of the Nosé–Hoover algorithm was used for molecular dynamics simulations in the *NPT* ensemble at *T* = 300 K and *P* = 0–380 MPa by using the DL_POLY version 4.10 software. 0.5 and 1 ps relaxation times were employed for thermo- and barostat, respectively.

For simplicity, we used rigid tubes. Atoms were fixed in their crystallographic positions to save the tube structure during the simulations. Due to fluctuations in pressure, the sizes of the tubes vibrated during runs. They were also slightly different (less than 3%) at different external pressures due to the scaling of the atomistic coordinates, according to the barostat algorithm. This effect mimics the compressibility of the actual materials.

The SPC/Fw flexible water model was adopted for simulations with a recommended spherical cutoff radius of 9 Å.^75^ The simulation results can, to some extent, be dependent on the choice of the force field. For example, using the TIP4P/2005 water model, Mochizuki^54^ observed a spontaneous square ice formation in AFI-type PSZ at 300 K, but crystallization was absent at elevated temperatures. This force field provides a qualitatively correct description of the relative energies of ice for bulk water^76^ but does not exclude artifacts for water under nanoconfinement. SPC-type force fields correspond well to experimental intrusion/extrusion pressures for PSZs,^24,32,77^ and the short cutoff radius is optimal for accelerating calculations in the DL_POLY 4 program.

The interactions between tube atoms were neglected. The parameters of Lennard-Jones interactions for water and tube atoms are the same: ε = 0.1554 kcal/mol and σ = 3.1655 Å. The tubes are artificial objects whose hydrophobicity is regulated to a large extent by electrostatic interactions between water and tube atoms. In the present work, these electrostatic interactions are absent, providing a larger hydrophobicity of the tubes than the hydrophobicity of PSZs where silicon and oxygen atoms have partial electric charges. The roles of framework flexibility, partial electric charges, and water models were discussed in previous publications (see Supporting Information).^32,77^ It was shown that the decrease of charges shifts intrusion–extrusion pressures toward higher values, thus making the material more hydrophobic.

The smooth particle mesh Ewald method was used to calculate the long-range part of the electrostatic interactions. Equations of motion were integrated numerically by the velocity Verlet method with a time step of 1 fs. The duration of the runs depended on the pressure and width of the tubes, reaching 20 ns in some cases. An example of DL_POLY files containing all information about the simulation method and force fields is presented in Supporting Information.

## Supporting Information Available

DL_POLY input files (ZIP)
Characteristics of pure silica zeolites and grafted mesoporous silica materials with the 1D system of channels; pore openings in zeolites with the 1D system of channels; evolution of dipole orientations in the water cluster in the tube; time evolutions of the average cosine and the number of water molecules in the tube; fragments of square ice structure; evolution of average cosine between dipole moments and the axial direction of the tube; collection of snapshots of clusters in the tube; extrusion of water from the tube; and statistics of H bonds for water in tubes (PDF)
