# Supplementary material for: Spontaneous Dipole Reorientation in Confined Water and Its Effect on Wetting/Dewetting of Hydrophobic Nanopores
Source: ACS Appl Mater Interfaces. 2024 Feb 1;16(6):7604–16. doi: 10.1021/acsami.3c17272 (PMC10875646; doi:10.1021/acsami.3c17272)
Supplement: Supplementary file 2 — am3c17272_si_002.pdf [file am3c17272_si_002.pdf]

SUPPORTING INFORMATION

Spontaneous dipole reorientation in confined water and its effect on wetting/dewetting of hydrophobic nanopores

Yuriy G. Bushuev,[a*] Yaroslav Grosu,[a,b] Mirosław Chorążewski [a]

[a]*Institute of Chemistry, University of Silesia in Katowice, Szkolna 9 street, 40-006 Katowice, Poland*

[b]*Centre for Cooperative Research on Alternative Energies (CIC energiGUNE), Basque Research and Technology Alliance (BRTA), Alava Technology Park, Albert Einstein 48, 01510 Vitoria-Gasteiz, Spain*

Email: yuriy.bushuev@us.edu.pl

Table S1. Characteristics of pure silica zeolites and grafted mesoporous silica materials with the 1D system of channels

Pore opening, *d*, Å	Intrusion pressure, MPa	Extrusion pressure, MPa	1/*d*, $Å^{-1}$	**Topology**
		Pure silica zeolites [a,b]		
10.09	75	75	0.13333	CFI
12.25[c]	26	21	0.11364	DON
6.75	125	125	0.15873	MTF
8.08	51	37	0.13158	STF
8.71	186	172	0.17544	TON
7.66	176	174	0.16129	MTT
8.49[c]	132	126	0.16447	MTW
10.06	57	55	0.12048	AFI
	Grafted mesoporous silica[1]			**Material**
26	59.5	30.8	0.03846	MTS-1g
32	44.4	6.2	0.03125	MTS-2g
40	35	2.5	0.025	MTS-3g
108	14.4	No extrusion	0.00926	MTS-4g
	Grafted mesoporous silica[2]			
26.8	43.21	17.8	0.03731	MCM-41
30.8	32.55	10.94	0.03247	HMS
43	29.11	6.02	0.02326	SBA-15
	Grafted mesoporous silica[3]			
60	17.5	No extrusion	0.01667	RPB

[a] Distances from Figure S1 (see the Database of Zeolite Structures, https://www.iza-structure.org/databases/).

[b] Intrusion and extrusion pressures from L. Ronchi, J. Patarin, H. Nouali, T. Jean Daou and A. Ryzhikov **Structure Influence on High-Pressure Water Intrusion in Pure Silica Zeolites.** ***New J. Chem.***, 2024, Accepted Manuscript. https://doi.org/10.1039/D3NJ03991A.

[c] The average value.

(1) Lefevre, B.; Saugey, A.; Barrat, J. L.; Bocquet, L.; Charlaix, E.; Gobin, P. F.; Vigier, G. Intrusion and Extrusion of Water in Hydrophobic Mesopores. *J. Chem. Phys.* **2004**, *120* (10), 4927–4938. https://doi.org/10.1063/1.1643728.

(2) Guillemot, L.; Biben, T.; Galarneau, A.; Vigier, G.; Charlaix, É. Activated Drying in Hydrophobic Nanopores and the Line Tension of Water. *Proc. Natl. Acad. Sci. U. S. A.* **2012**, *109* (48), 19557–19562. https://doi.org/10.1073/pnas.1207658109.

(3) Amabili, M.; Grosu, Y.; Giacomello, A.; Meloni, S.; Zaki, A.; Bonilla, F.; Faik, A.; Casciola, C. M. Pore Morphology Determines Spontaneous Liquid Extrusion from Nanopores. *ACS Nano* **2019**, *13* (2), 1728–1738. https://doi.org/10.1021/acsnano.8b07818.

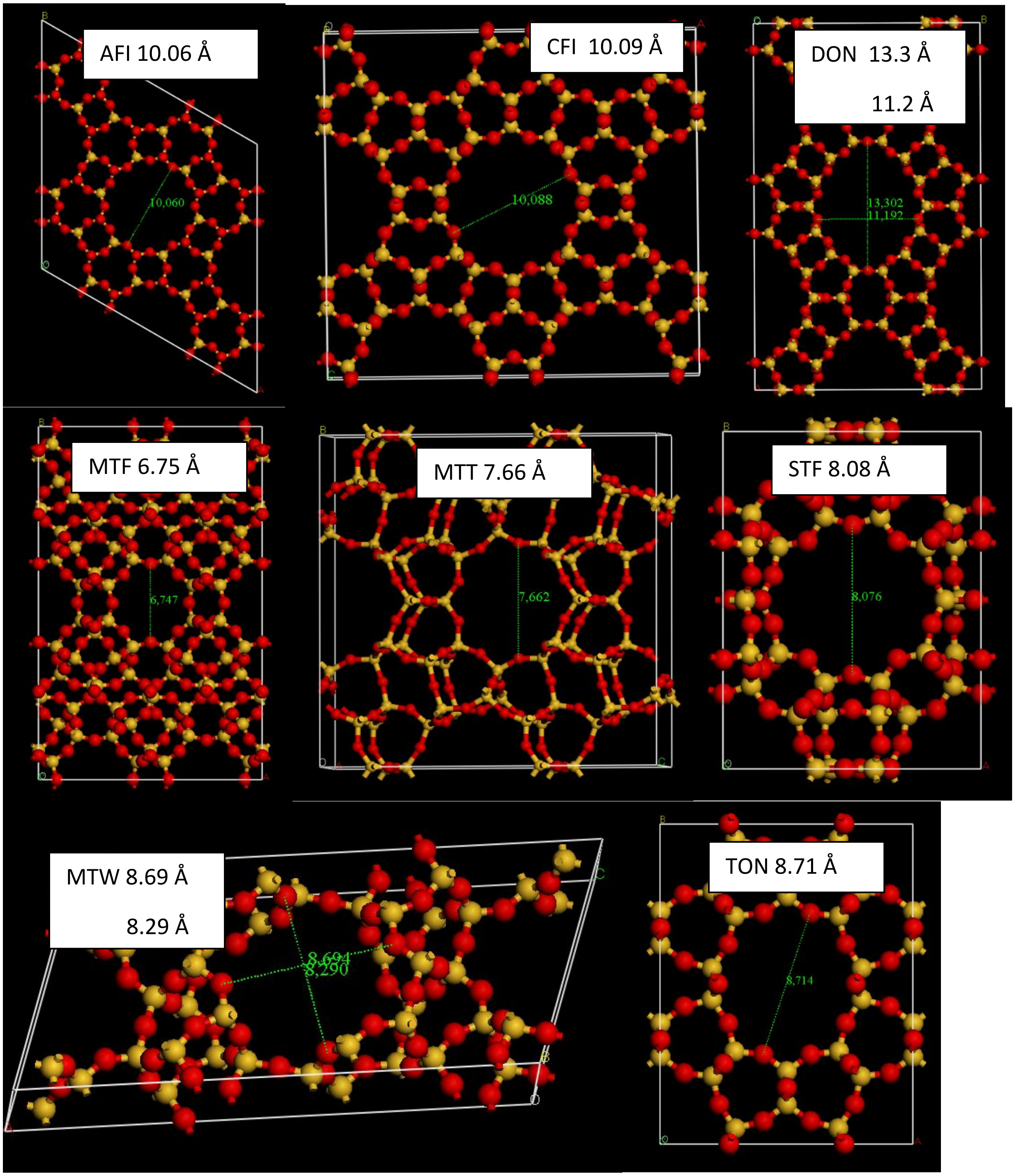

Figure S1. Pore openings in zeolites with the 1D system of channels. The cif files are presented in the Database of Zeolite Structures, https://www.iza-structure.org/databases/.

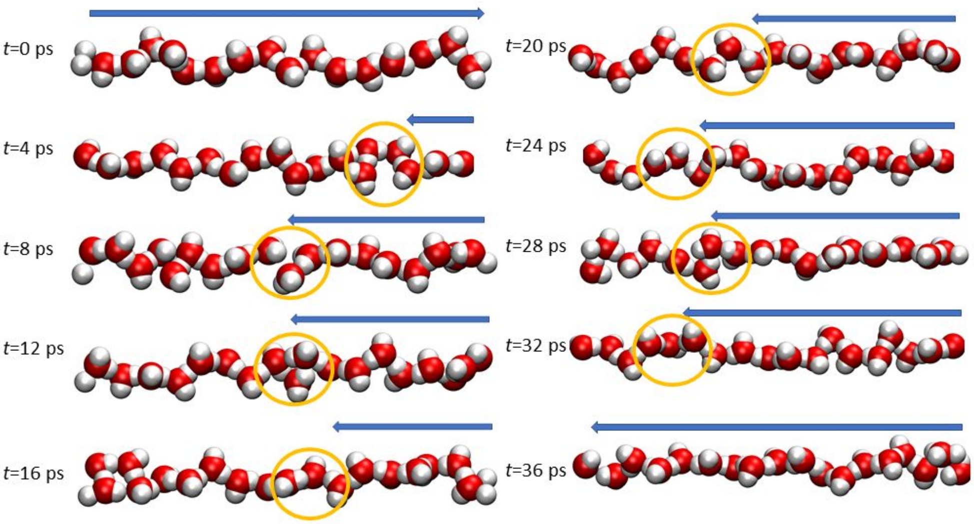

Figure S2. The evolution of dipole orientations in the water cluster in the tube (d = 7.9 Å, P = 380 MPa) corresponds to the flipping event #4 in Figure 3b. Arrows show the orientation of dipole moments in the initial or final chains and the propagating reoriented fragment. Ellipses highlight positions of orientational defects.

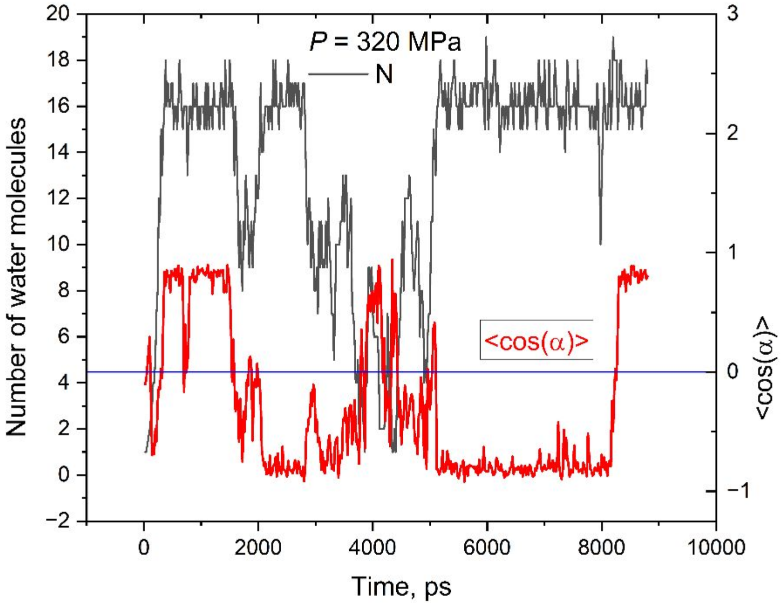

Figure S3. Time evolutions of the average cosine and the number of water molecules in the tube (d = 7.9 Å) at P = 320 MPa.

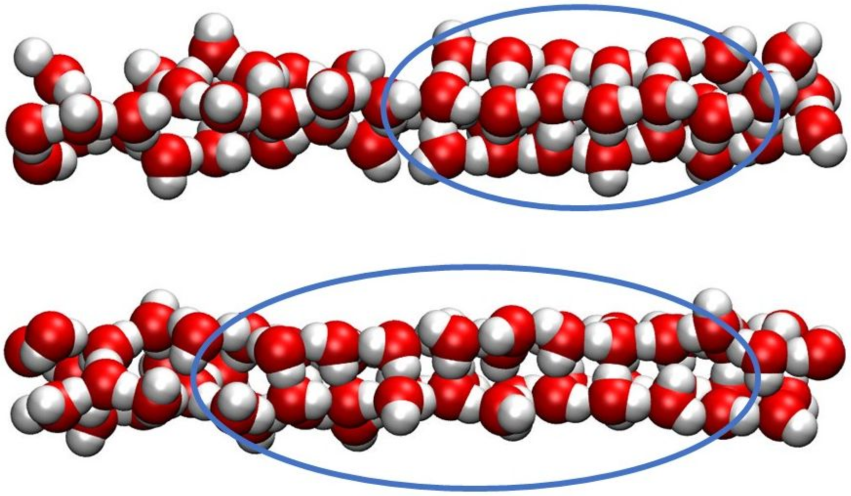

Figure S4. Fragments of square-ice structure (highlighted by ellipses) in the tube with d=10 A at P=170 MPa.

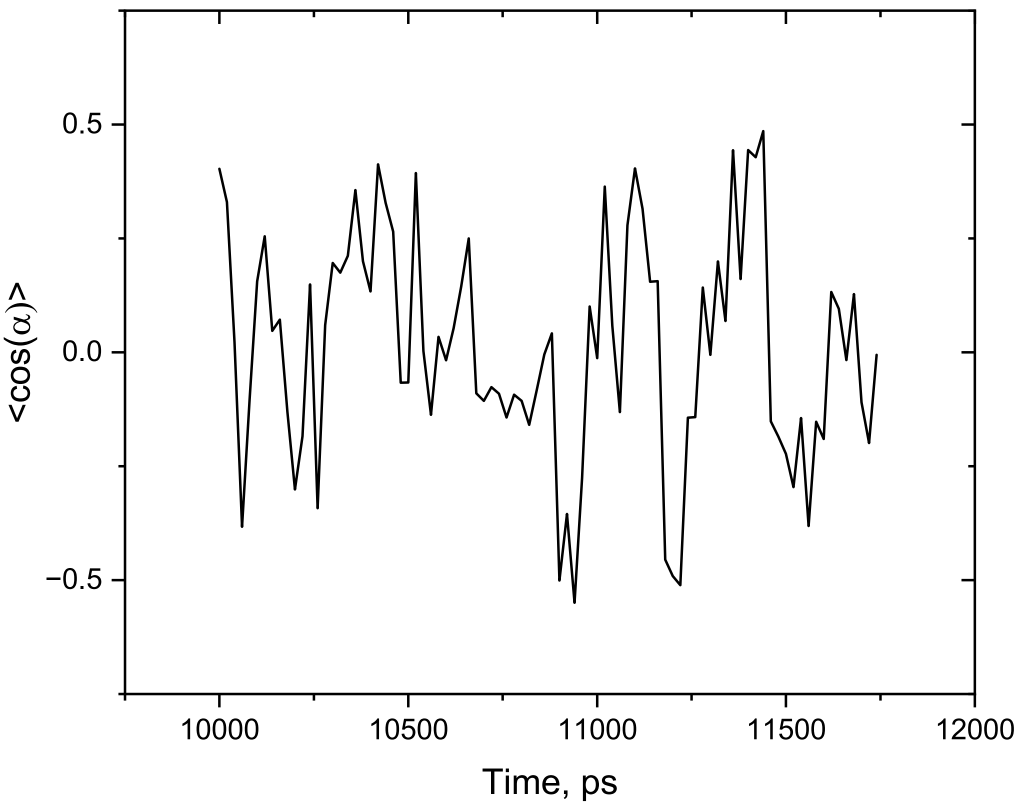

Figure S5. Evolution of average cosine between dipole moments and the axial direction of the tube with *d*=10 A at *P*=180 MPa.

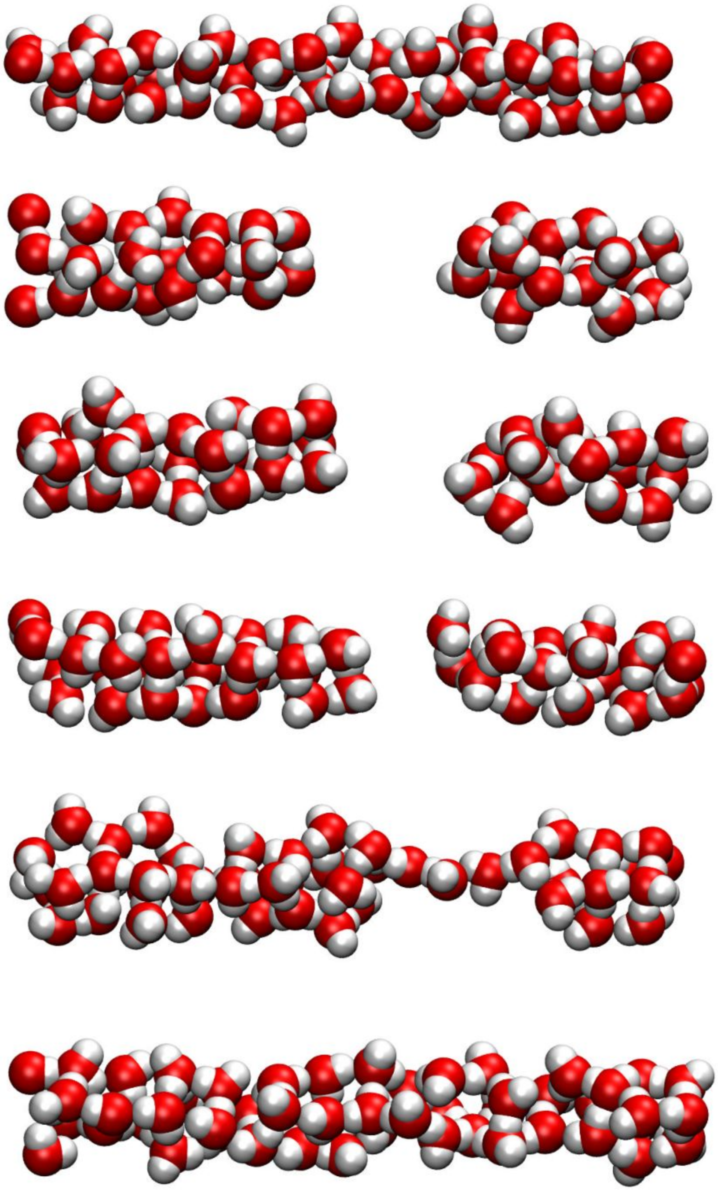

Figure S6. Snapshots demonstrate the evolution of water clusters in the tube with *d*=10 A at *P*=170 MPa. The timestep is 20 ps.

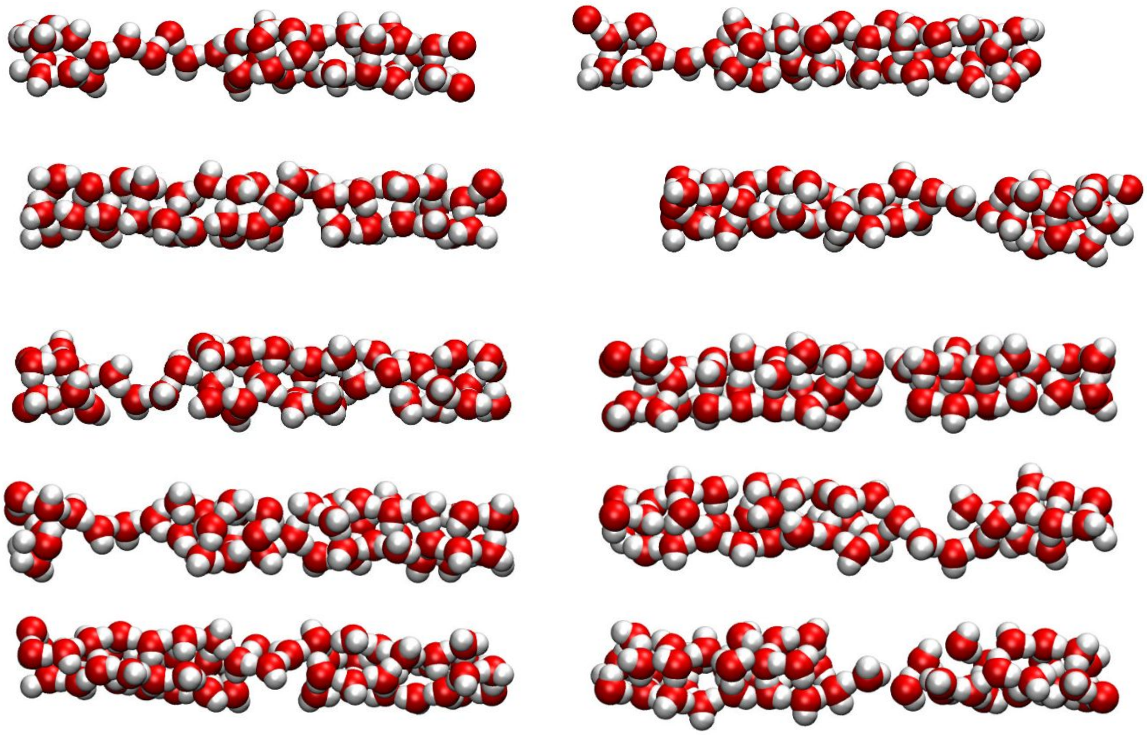

Figure S7. The collection of snapshots of clusters in the tube with *d*=10 A that are close to being split.

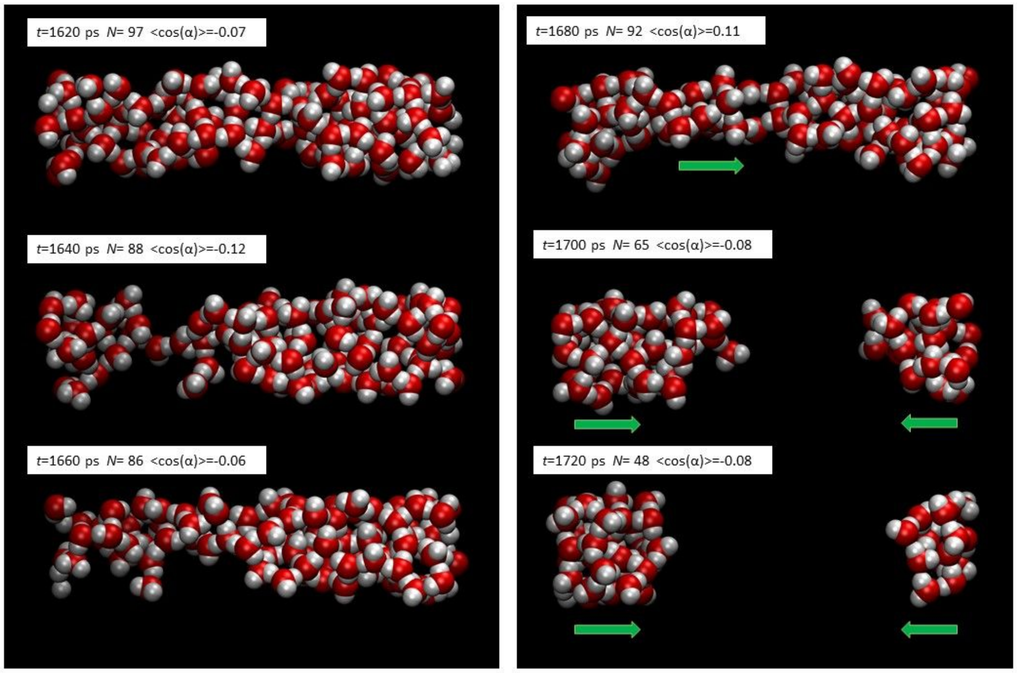

Figure S8. Extrusion of water from the tube with *d*=14.3 Å at *P*=10 MPa. Arrows show the preferential orientation of dipole moments, *t* is the time mark, *N* is the number of water molecules in the tube.

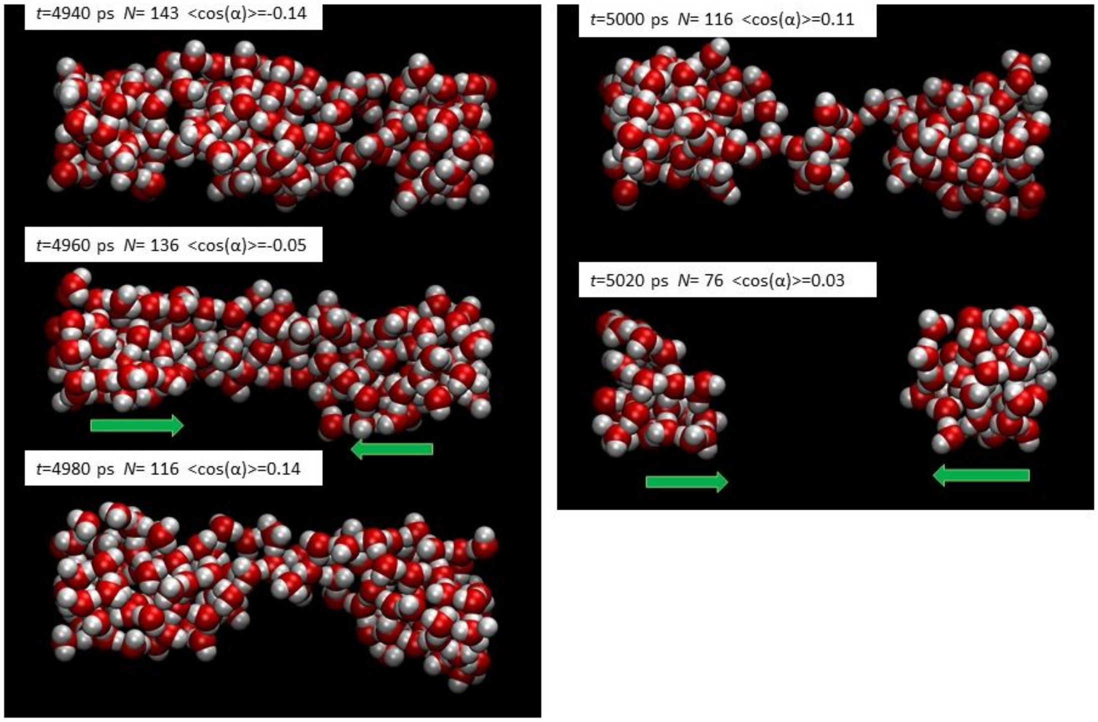

Figure S9. Extrusion of water from the tube with *d*=16.5 Å at *P*=-20 MPa. Arrows show the preferential orientation of dipole moments, *t* is the time mark, *N* is the number of water molecules in the tube.

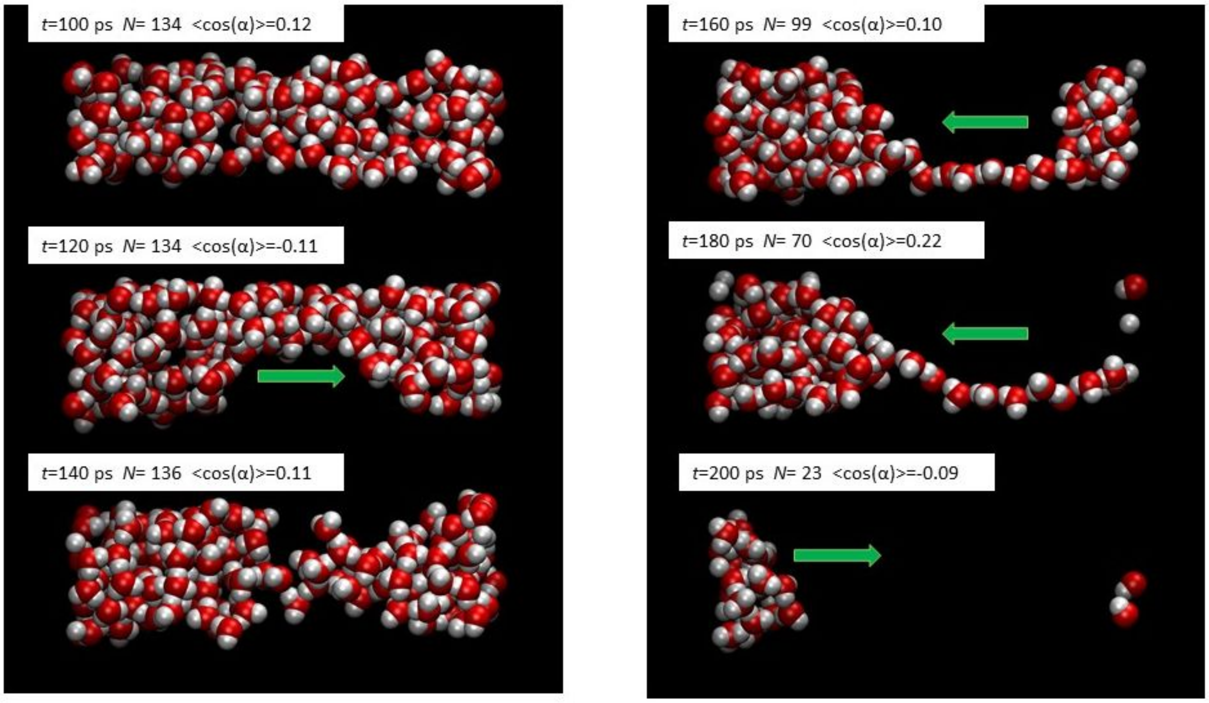

Figure S10. Extrusion of water from the tube with *d*=16.5 Å at *P*=-30 MPa. Arrows show the preferential orientation of dipole moments, *t* is the time mark, *N* is the number of water molecules in the tube.

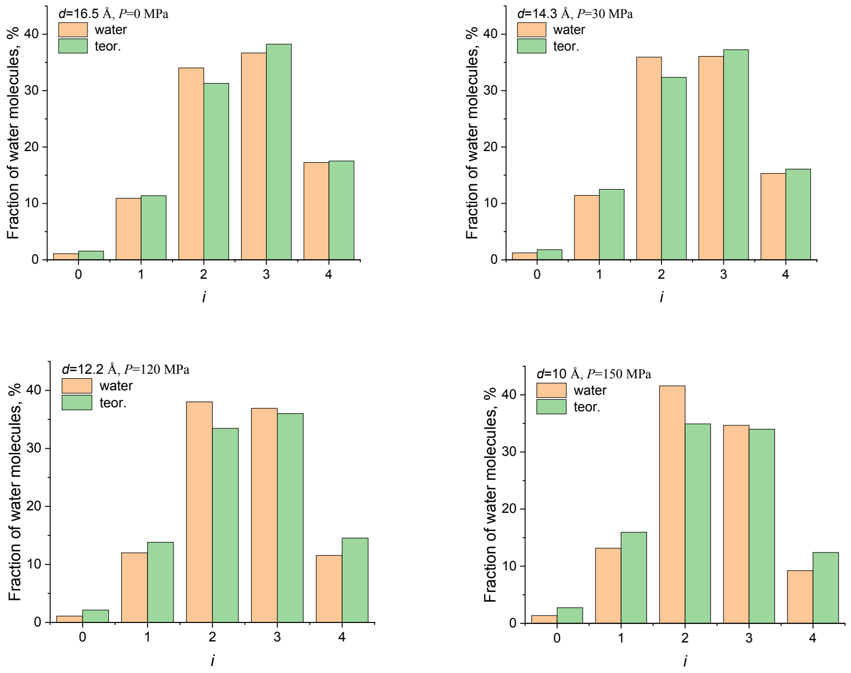

Figure S11. Statistics of H-bonds for water in tubes, comparing with theoretical predictions, Eq 2.

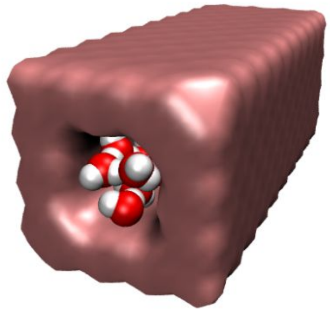

Figure S12. The tube (d = 10 Å) is inserted into the simulation box filled with water. Only water molecules in the tube are shown for clarity.
